# Dynamic estimates of survival of patients with poorly differentiated thyroid carcinoma: a population-based study

**DOI:** 10.3389/fendo.2024.1375274

**Published:** 2024-09-13

**Authors:** Zhao Liu, Qianlan Xu, Heng Xia, Miaofeng Wang

**Affiliations:** ^1^ Department of Breast and Thyroid Surgery, Shaoxing Central Hospital, The Central Affiliated Hospital, Shaoxing University, Shaoxing, China; ^2^ Department of gynecology, Shaoxing Central Hospital, The Central Affiliated Hospital, Shaoxing University, Shaoxing, China

**Keywords:** poorly differentiated thyroid carcinoma, SEER, prognosis, conditional survival, nomogram

## Abstract

**Background:**

The real-time prognostic data of patients with poorly differentiated thyroid carcinoma (PDTC) after surviving for several years was unclear. This study aimed to employ a novel method to dynamically estimate survival for PDTC patients.

**Methods:**

A total of 913 patients diagnosed with PDTC between 2014 and 2015 from the Surveillance, Epidemiology, and End Results (SEER) database, was recruited in our study. Kaplan–Meier method was used to estimate the overall survival (OS). The conditional survival (CS) outcomes of PDTC were analyzed and CS rates were calculated using the formula CS(y/x) = OS(y+x)/OS(x), whereby CS(y/x) denotes the probability of a patient enduring an additional y years subsequent to surviving x years following the diagnosis of PDTC. The least absolute shrinkage and selection operator (LASSO) regression was employed to identify prognostic predicters and multivariate Cox regression was utilized to develop a CS-nomogram. Finally, the performance of this model was evaluated and validated.

**Results:**

Kaplan–Meier survival analysis unveiled patient outcomes demonstrating an OS rate of 83%, 75%, and 60% respectively at the end of 3, 5, and 10 years. The novel CS analysis highlighted a progressive enhancement in survival over time, with the 10-year cumulative survival rate progressively augmenting from its initiation of 60% to 66%, 69%, 73%, 77%, 81%, 83%, 88%, 93%, and finally 97% (after surviving for 1-9 years, respectively) each year. And then 11 (11/15) predictors including age at diagnosis, sex, histology type, SEER stage, T stage, N stage, M stage, tumor size, coexistence with other malignancy, radiotherapy and marital status, were selected by LASSO analysis under the condition of lambda.min. Multivariate Cox regression analysis further highlighted the significant impact of all these predictors on the OS of PDTC and we successfully established and validated a novel CS-nomogram for real-time and dynamic survival prediction.

**Conclusions:**

This was the first study to analyze the CS pattern and demonstrate a gradual improvement in CS over time in long-term PDTC survivors. We then successfully developed and validated a novel CS-nomogram for individualized, dynamic, and real-time survival forecasting, empowering clinicians to adapt and refine the patient-tailored treatment strategy promptly with consideration of evolving risks.

## Introduction

Poorly differentiated thyroid carcinoma (PDTC), a predominantly rare clinical entity, comprises approximately 2-15% of the total diagnosed thyroid cancers (1–4). The term of PDTC emerged initially during the 1980s, yet its definitive acknowledgement as a unique pathological classification did not occur until the issuance of the most recent version of the Endocrine Tumor Classification system by the World Health Organization (WHO) in 2004 (5). As defined by the WHO classification, PDTC possesses a unique natural course and histopathological traits which establish it as an intermediary entity between well-differentiated and undifferentiated (anaplastic) thyroid malignancies (6). And in these tumors, advanced stage metastases are prevalent, with lung and bone representing the most frequent sites of disseminated metastases (7). Owing to its aggressive characteristics, PDTC stands as the predominant contributor to cancer-specific mortality within non-anaplastic follicular cell-derived thyroid carcinomas, signifying an urgent demand for further research on this disease.

With advancements in screening methodology and treatment strategy, the survival of these PDTC patients has improved significantly, demonstrating 5- and 10-year overall survival (OS) rates of 70% and 50%, respectively (8, 9). Nevertheless, these survival data offer solely a restricted predictive value for new individuals who receive their diagnoses. And long-term survivors may exhibit heightened concern regarding precise information on survival evaluation. However, traditional survival evaluations, estimated from the time of diagnosis, failed to provide accurate survival predictions for individuals who maintained survival for multiple years. Thus, utilization of an innovative prognostication computation methodology is imperative to supply precise and real-time survival data for these patients.

Conditional survival (CS), which was defined as the probability that a patient would survive for a further β years after surviving for α years after diagnosis, could calculate real-time survival data and provide continuously updated predictive insights for cancer patients (10–12). And CS analysis, compared to traditional survival methods like Kaplan-Meier estimates, offers the unique ability to provide dynamic survival probabilities that evolve over time, reflecting the changing risk for patients who have already survived a certain period post-diagnosis. And this novel computational methodology has been extensively employed across several cancer types (13–16); however, the CS outcomes for PDTC remains unexplored.

Therefore, the aim of the present study was to analyze the CS of PDTC patients and propose a novel CS-integrated nomogram model for dynamic prognosis prediction based on the latest version of the Surveillance, Epidemiology and End Results (SEER) database.

## Methods

### SEER data source, patients and clinical variables

The SEER database, which covers approximately 34.6% of the population of the United States, possessing prospective data collection, obviated the necessity for institutional review board approval for this research. PDTC cases diagnosed between 2004 and 2015 were extracted from the SEER database using SEER*Stat (version 8.3.8) software. A total of 913 poorly differentiated grade III samples recognized with a pathologically verified PDTC as defined by the International Classification of Diseases for Oncology 3rd Edition (ICD-O-3) site classification, encompassing papillary thyroid carcinoma (PTC, 8050/3, 8260/3, and 8340/3), follicular thyroid carcinoma (FTC, 8330/3), and insular thyroid carcinoma (ITC, 8337/3).

Variables of interest included age at diagnosis (aged ≤40, 41-60 and >60 years), sex, race (white and nonwhite), histology type (PTC, FTC and ITC), SEER stage (localized, regional and distant), tumor size (<20mm, 20-39mm, and ≥40mm), coexistence with other malignancy, T stage (T1, T2, T3 and T4), N stage (N0 and N1), M stage (M0 and M1), as well as surgical procedures performed (non-total thyroidectomy and total thyroidectomy), receipt of radiotherapy treatment (no and yes), marital status categories (single, married and unknown), rural-urban (nonmetropolitan and metropolitan) and household income (<$65000 and ≥$65000). In the context of the SEER database, the definition for radiotherapy relates exclusively to treatment employing beam radiation, radioiodine, or a blend of the aforementioned.

### Statistical analysis

The primary endpoint of this study was OS, with designated duration being the period extending from the time of initial diagnosis until either mortality or the final documented follow-up appointment. Categorical data was presented in the form of counts and associated percentages.

#### Conditional survival analysis

The Kaplan-Meier method was used to estimate OS and absolute hazard ratio. The CS rate was calculated using the formula CS(y/x) = OS(y+x)/OS(x), whereby CS(y/x) denotes the probability of a patient enduring an additional y years subsequent to surviving x years following the diagnosis of PDTC; and OS(y/x) and OS(x), respectively refer to the (y/x)-and x-year OS rates estimated via the Kaplan-Meier approach.

#### Annual hazard rate

The annual hazard was computed by subdividing temporal duration into distinct sections over years, then determining the mortality statistic independently for each section, i.e., annual hazard rate=the count of casualties within a year/cumulative follow-up patients in the year (17).

#### CS-nomogram development

To establish the CS-nomogram, the identified patients were haphazardly stratified into the training and testing cohorts in a proportionate ratio of 7 to 3. For the predictor selection, we employed the Least Absolute Shrinkage and Selection Operator (LASSO) regression technique in conjunction with a stringent 10-fold cross-validation protocol, thereby mitigating the potential drawbacks of model overfitting. The filtered predictive indicators were elucidated regarding their influence on PDTC survival via multivariate Cox regression, subsequently utilized in the development of a CS-nomogram model. Our novel research endeavor incorporated the CS formula into the bespoke nomogram to allow for personalized predictors and dynamic survival estimation for individuals afflicted by PDTC.

#### Risk classification system construction

The CS-nomogram quantified these predictive factors as points, where incorporating the patient’s unique variables could culminate into a total point score. Then we developed a risk classification system based on the optimal threshold of point scores among all patients to execute risk stratification for these patients. The Kaplan–Meier method with a log-rank test was conducted to evaluate the discrepancies in OS across different risk groups.

#### Prediction model evaluation and validation

Calibration plots were employed for comparative evaluation of the projected survival outcomes from our nomogram against the observed outcome data. The discriminatory power of the CS-nomogram was critically evaluated via varies methodologies, encompassing the concordance index (C-index), and receiver operating characteristic (ROC) curves paired with the area under this curve (AUC). The C-index is a metric used to assess the predictive accuracy of survival models. The C-index value ranges from 0.5 to 1, with values closer to 1 indicating stronger predictive capability of the model. And the time-dependent ROC curve allows for the assessment of a model’s predictive performance at various time points. By examining these curves, one can evaluate how well the model performs throughout the follow-up period and discern whether it excels in early or late predictions. Moreover, the efficacy of the clinical application of this prediction model was verified through Decision Curve Analysis (DCA), which quantified the potential net advantage garnered by utilizing the nomogram.

This study’s statistical analysis was conducted utilizing the R (version 4.1.0), with a significance level set at P values inferior to 0.05 for binary analyses.

## Results

### Demographic and clinicopathological features

We incorporated clinical data of 913 individuals diagnosed with PDTC from the SEER database within our analysis, randomizing them into two distinct cohorts, including a training group and testing group at a ratio of 7:3. Among all individuals diagnosed with PDTC, those over the age of 60 represented 44.0%, females comprised 61.2%, and a substantial 77.9% were white. Concerning the clinical pathological attributes, the vast majority of PDTCs were of the PTC subtype (69.3%), exceeding 40mm in size (47.6%) and exhibiting a localized/regional stage (81.2%). Regarding treatment, the vast majority underwent total thyroidectomy (88.8%) and radiotherapy in 69.8% of patients. Detailed information on these PDTC patients is depicted in **Table 1**.

**Table 1:** Patient clinicopathologic characteristics in PDTC.

Variables	All (N=913)	Training (N=639)	Validation (N=274)
Age at diagnosis			
≤40	167 (18.3%)	113 (17.7%)	54 (19.7%)
41-60	344 (37.7%)	244 (38.2%)	100 (36.5%)
>60	402 (44.0%)	282 (44.1%)	120 (43.8%)
Sex			
Male	354 (38.8%)	240 (37.6%)	114 (41.6%)
Female	559 (61.2%)	399 (62.4%)	160 (58.4%)
Race			
White	711 (77.9%)	492 (77.0%)	219 (79.9%)
Nonwhite	202 (22.1%)	147 (23.0%)	55 (20.1%)
Histology type			
PTC	633 (69.3%)	440 (68.9%)	193 (70.4%)
FTC	132 (14.5%)	95 (14.9%)	37 (13.5%)
ITC	148 (16.2%)	104 (16.3%)	44 (16.1%)
SEER stage			
Localized	389 (42.6%)	272 (42.6%)	117 (42.7%)
Regional	352 (38.6%)	242 (37.9%)	110 (40.1%)
Distant	172 (18.8%)	125 (19.6%)	47 (17.2%)
Tumor size			
<20	189 (20.7%)	145 (22.7%)	44 (16.1%)
20-39	289 (31.7%)	197 (30.8%)	92 (33.6%)
≥40	435 (47.6%)	297 (46.5%)	138 (50.4%)
Coexistence with other malignancy			
No	656 (71.9%)	451 (70.6%)	205 (74.8%)
Yes	257 (28.1%)	188 (29.4%)	69 (25.2%)
T			
T1	149 (16.3%)	110 (17.2%)	39 (14.2%)
T2	167 (18.3%)	119 (18.6%)	48 (17.5%)
T3	417 (45.7%)	279 (43.7%)	138 (50.4%)
T4	180 (19.7%)	131 (20.5%)	49 (17.9%)
N			
N0	614 (67.3%)	437 (68.4%)	177 (64.6%)
N1	299 (32.7%)	202 (31.6%)	97 (35.4%)
M			
M0	808 (88.5%)	561 (87.8%)	247 (90.1%)
M1	105 (11.5%)	78 (12.2%)	27 (9.9%)
Surgery			
Non-total thyroidectomy	102 (11.2%)	74 (11.6%)	28 (10.2%)
Total thyroidectomy	811 (88.8%)	565 (88.4%)	246 (89.8%)
Radiotherapy			
No	276 (30.2%)	186 (29.1%)	90 (32.8%)
Yes	637 (69.8%)	453 (70.9%)	184 (67.2%)
Marital status			
Single	354 (38.8%)	245 (38.3%)	109 (39.8%)
Married	521 (57.1%)	369 (57.7%)	152 (55.5%)
Unknown	38 (4.2%)	25 (3.9%)	13 (4.7%)
Rural-Urban			
Nonmetropolitan	85 (9.3%)	62 (9.7%)	23 (8.4%)
Metropolitan	828 (90.7%)	577 (90.3%)	251 (91.6%)
Household income			
<$65000	409 (44.8%)	290 (45.4%)	119 (43.4%)
≥$65000	504 (55.2%)	349 (54.6%)	155 (56.6%)

PDTC, poorly differentiated thyroid carcinoma. PTC, papillary thyroid carcinoma; FTC, follicular thyroid carcinoma; ITC, insular thyroid carcinoma.

### Conditional survival and annual hazards over time in PDTC patients

**Figure 1:**
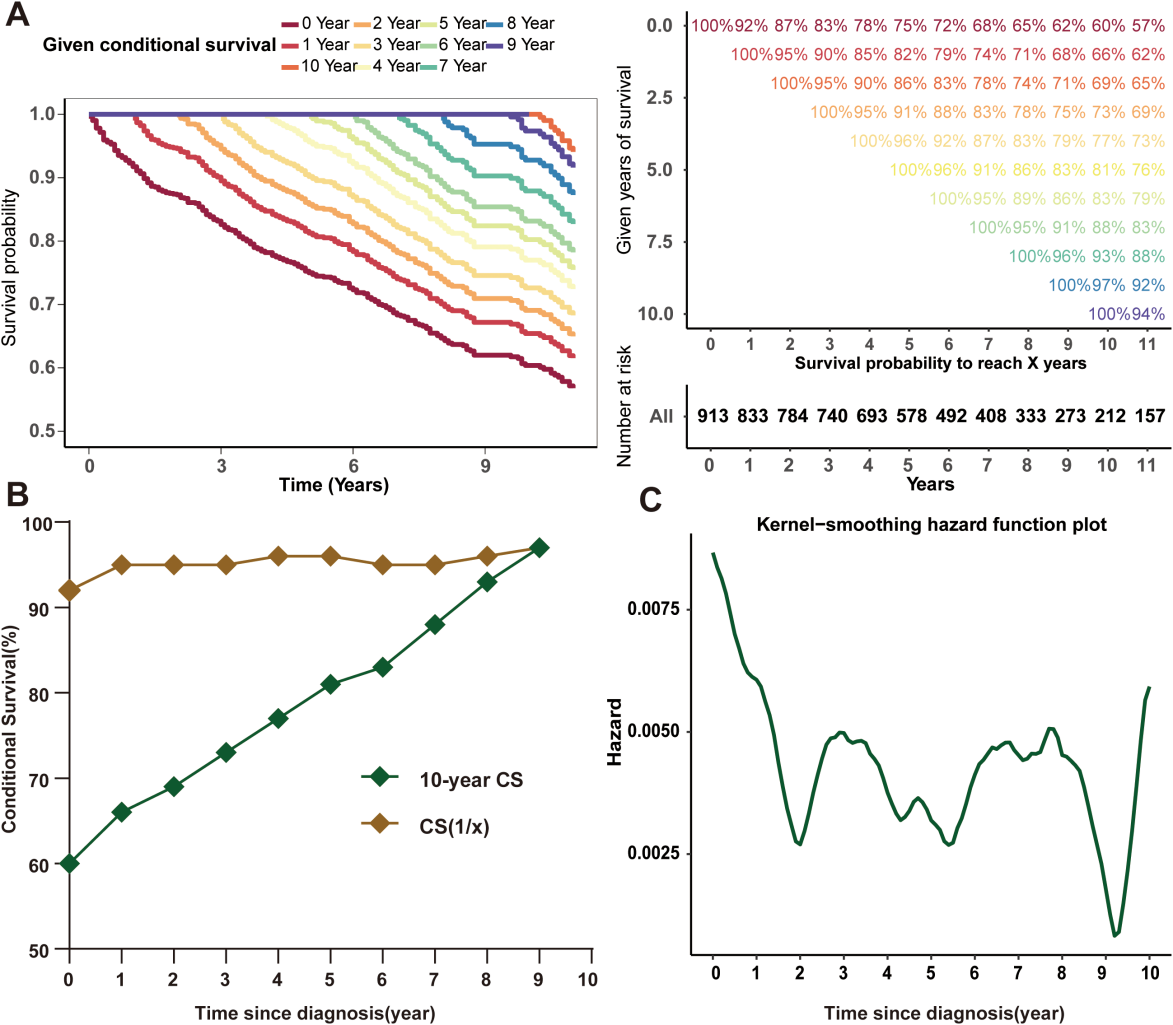
Conditional survival of PDTC patients. Kaplan-Meier method for estimating conditional survival after surviving for 0-9 years **(A)**; 10-year conditional survival curves for PDTC patients and the survival rate curve for the next year after surviving a certain time **(B)**; Annual hazard rate curve **(C)**.

Traditional survival analysis unveiled patient outcomes demonstrating an OS rate of 83%, 75%, and 60% respectively at the end of 3, 5, and 10 years. The novel CS analysis highlighted a progressive enhancement in survival over time, with the 10-year cumulative survival rate progressively augmenting from its initiation of 60% to 66%, 69%, 73%, 77%, 81%, 83%, 88%, 93%, and finally 97% (after surviving for 1-9 years, respectively) each year (**Figure 1A**). The CS(1|x) curve, demonstrating the probability of patients enduring multiple years preceding an additional year of survival, showed graphically a potentially substantial enhancement in survival within the initial year post-diagnosis (**Figure 1B**). Moreover, the annual hazard curve elucidated the non-linear enhancement in PDTC survival over time by evaluating the instantaneous mortality of survivors and the **Figure 1C** showed the annual mortality rate of PDTC progressively declined from the initial diagnosis.

### Prediction model variable screening and nomogram construction

**Figure 2:**
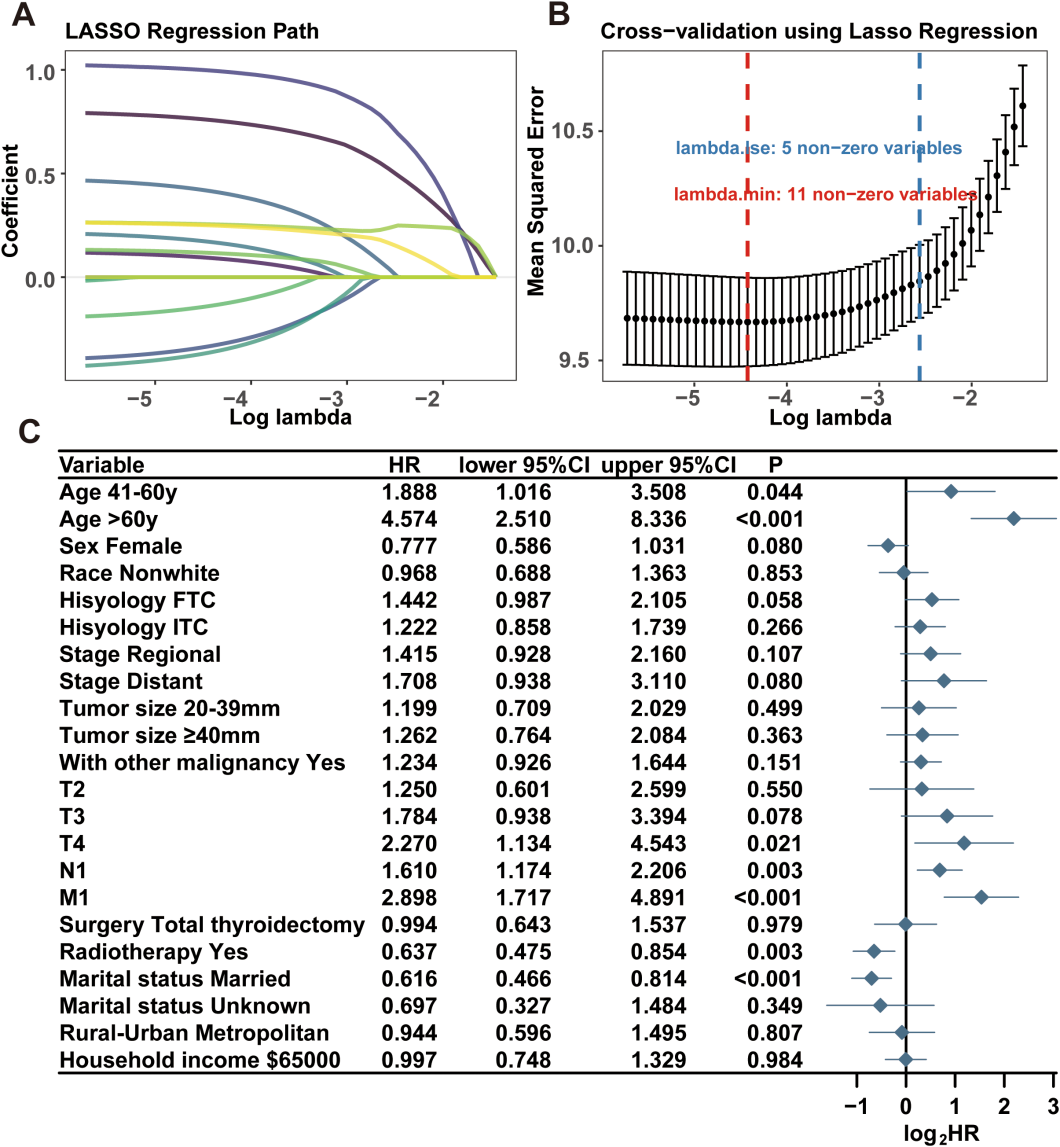
Prognostic factors identification. The least absolute shrinkage and selection operator (LASSO) regression for screening prognostic factors **(A, B)**. Multivariate Cox regression forest plot showing the effect of predictors on overall survival of PDTC **(C)**. HR, hazard ratio; PDTC, poorly differentiated thyroid carcinoma; PTC, papillary thyroid carcinoma; FTC, follicular thyroid carcinoma; ITC, insular thyroid carcinoma.

**Figure 3:**
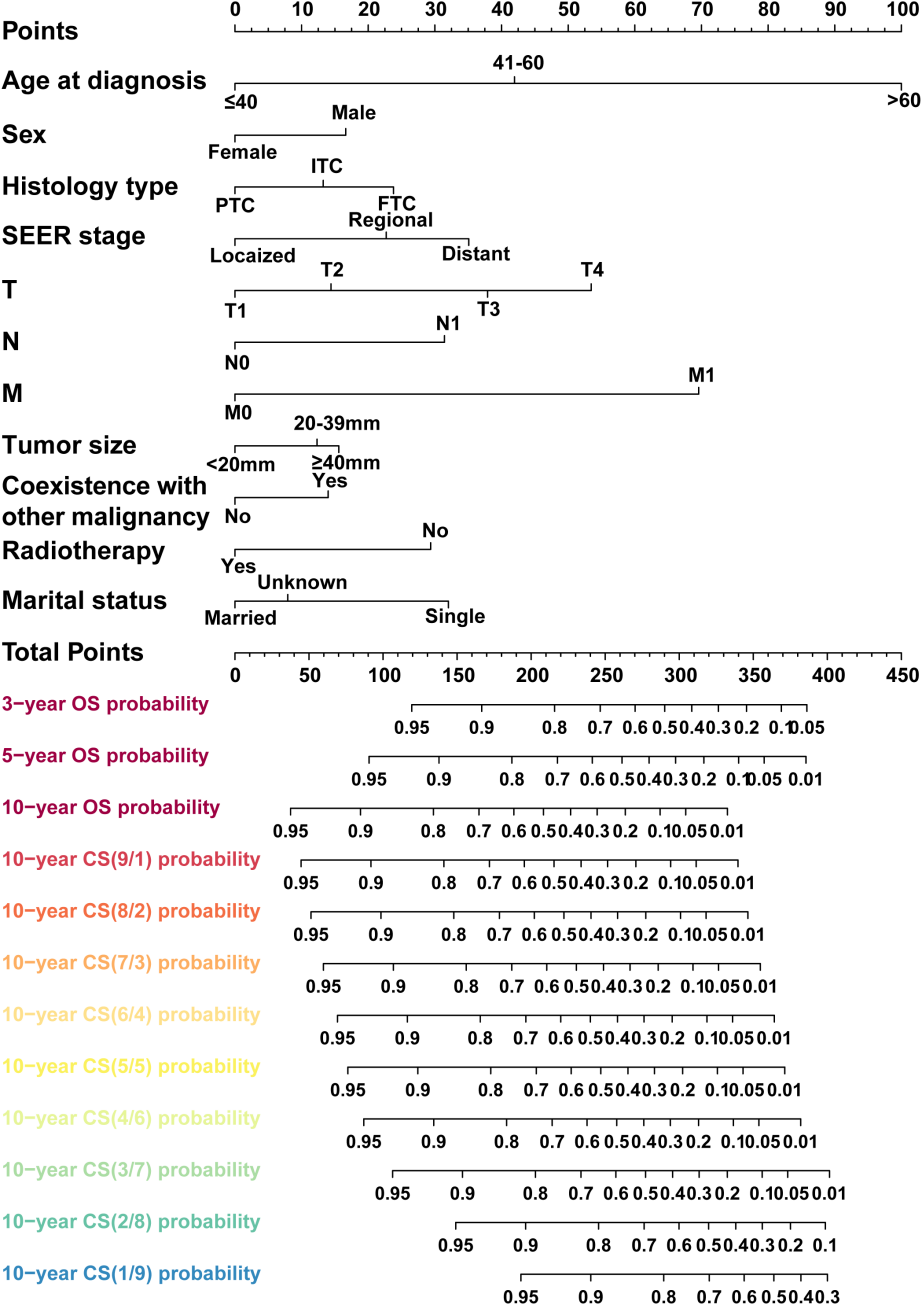
A conditional survival-based nomogram model was successfully developed for predicting 10-year CS for PDTC patients. PDTC, poorly differentiated thyroid carcinoma. PTC, papillary thyroid carcinoma; FTC, follicular thyroid carcinoma; ITC, insular thyroid carcinoma.

We then carried out the LASSO regression with 10-fold cross-validation in training cohort to screen prognostic factors for CS-nomogram construction. Ultimately, 11 (11/15) predictors including age at diagnosis, sex, histology type, SEER stage, T stage, N stage M stage, tumor size, coexistence with other malignancy, radiotherapy and marital status, were selected by LASSO analysis under the condition of lambda.min (**Figures 2A, B**). In LASSO regression, lambda.min is determined as the value of lambda that minimizes the cross-validated prediction error. And multivariate Cox regression analysis further highlighted the significant impact of all these predictors on the OS of PDTC (**Figure 2C**, P<0.05) and was used to develop a CS-nomogram model. Compared to traditional survival prediction models, the CS-nomogram utilized in this study yielded CS predictions, enabling patients to ascertain not only 3-, 5- and 10-year OS after inputting personalized clinicopathological factors but also 10-year CS contingent upon the number of years they had survived post-diagnosis (**Figure 3**).

### CS-nomogram evaluation and validation

**Figure 4:**
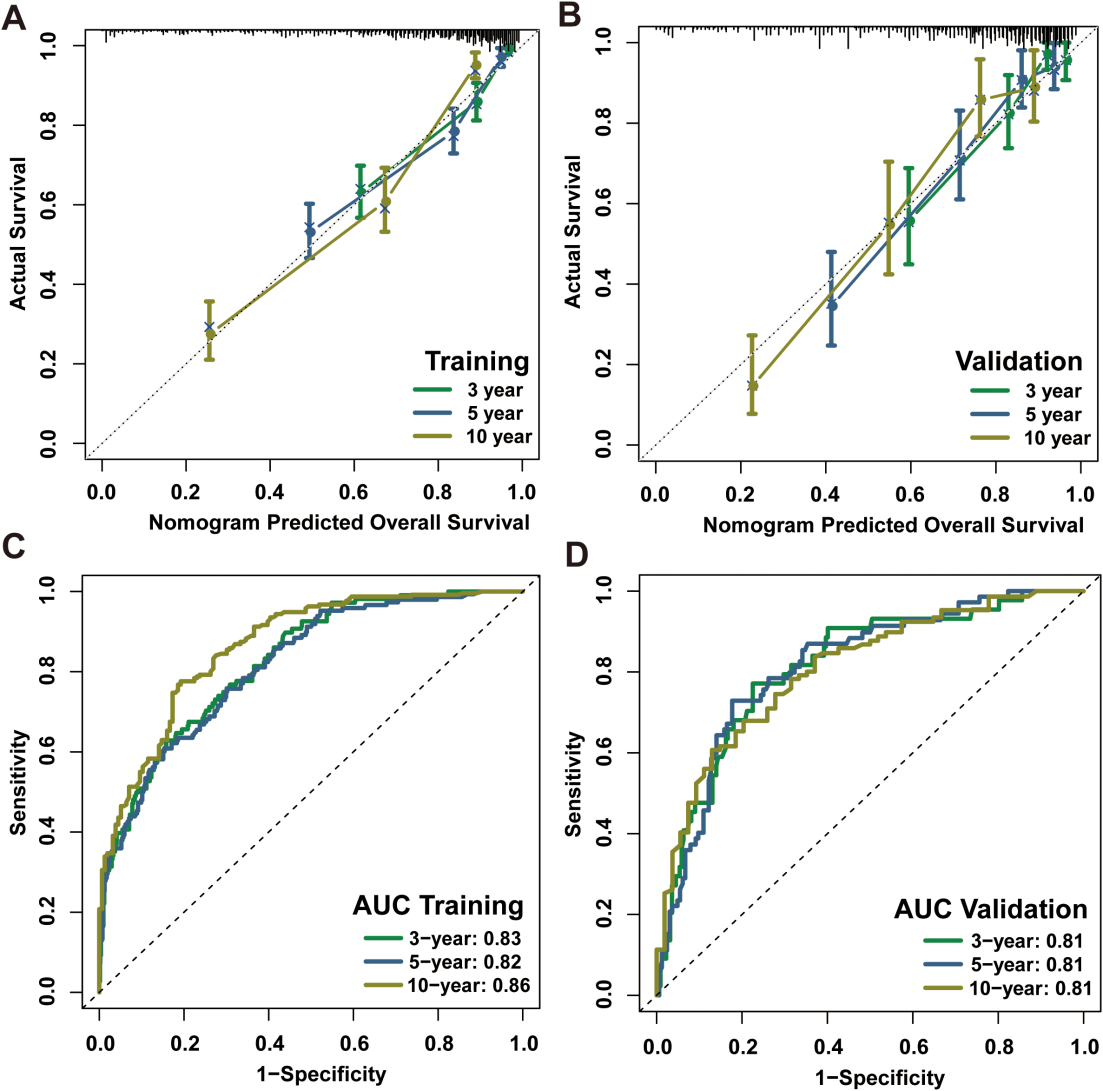
Calibration plots and ROC curves with AUC values for the CS-nomogram at 3, 5 and 10 years. Calibration plots of the CS-nomogram in training **(A)** and validation **(B)** cohorts; ROC curves of the CS-nomogram in training **(C)** and validation **(D)** cohorts. AUC, areas under the ROC curve; ROC, receiver operating characteristic curve; CS, conditional survival.

**Figure 5:**
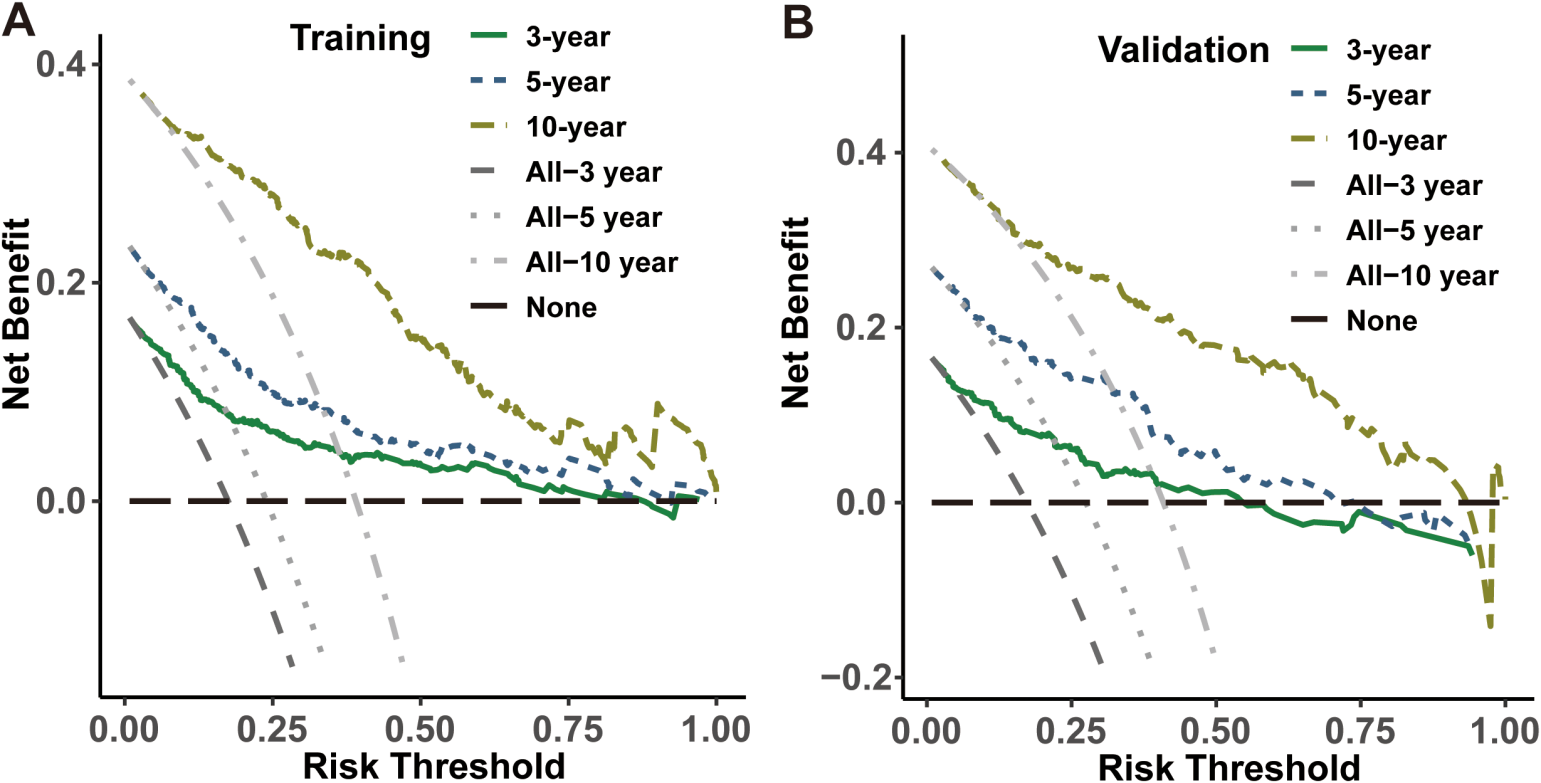
Decision curve analysis for the CS-nomogram in both the training **(A)** and validation **(B)** cohorts.

The present investigation evaluated the performance of the model in regards to accuracy, discrimination and utility. The C-index was applied to quantify the prediction ability of the nomogram model, yielding respective values of 0.798 and 0.703 in internal training and validation cohorts. Subsequently, nomogram calibration curves were depicted for 3-year, 5-year and 10-year intervals within each cohort, indicating a substantial consistency between anticipated and actual survival probabilities in both cohorts (**Figures 4A, B**). Besides, the time-dependent ROC at 3-, 5-, and 10-year AUC values in predicting CS were 0.83, 0.82, and 0.86 for the training set as well as 0.81, 0.81 and 0.81 for the validation set, respectively (**Figures 4C, D**). These results demonstrated the model had strong and consistent predictive performance across different time points, with stable AUC values in both the training and validation sets, indicating its robustness and reliable long-term predictive capability. Ultimately, DCA analysis was employed to evaluate the demonstrable clinical utility and advantages of the CS-nomogram in terms of predicting CS for PDTC patients. And these analyses at 3, 5 and 10 years demonstrated elevated net benefits across a range of mortality risks in both training and validation cohort for our novel CS-nomogram (**Figures 5A, B**), confirming the practical value of this clinical tool.

### Risk stratification system development

**Figure 6:**
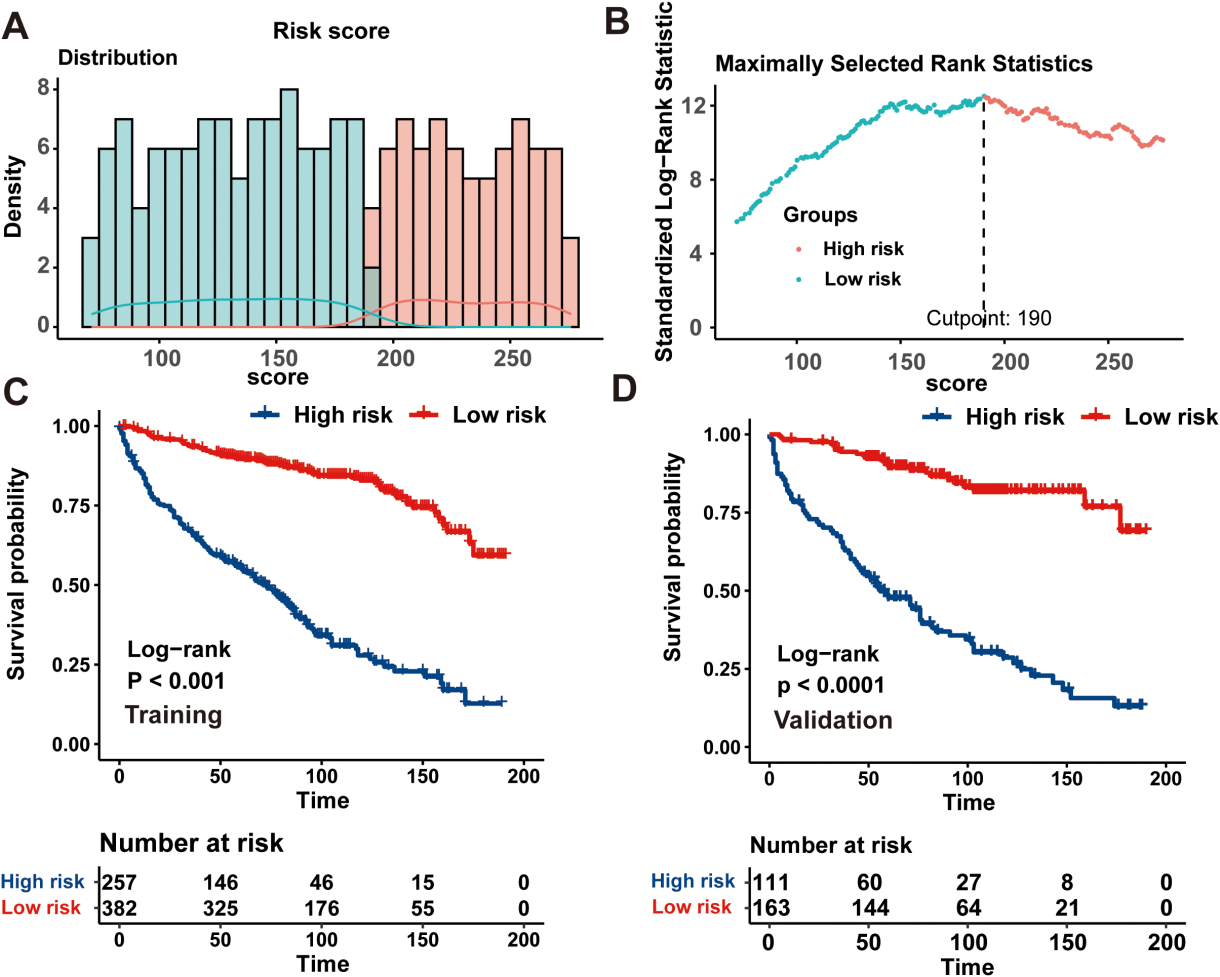
Establishment of a risk stratification system based on the condition survival- nomogram. **(A)** Distribution of risk points; **(B)** The standardized log-rank statistics; **(C, D)** Kaplan-Meier analysis utilizing the log-rank test for disparate risk groups across both training and validation cohorts.

Finally, a risk stratification system based on the CS-nomogram-derived scores was successfully developed. The CS-nomogram model was capable of quantifying all integrated variables as points and we calculated risk score for each individual. Then, based on the identified optimal threshold of 190, included patients were divided into low and high-risk groups (**Figures 6A, B**). Further analysis in the training and validation cohorts revealed that, for PDTC patients, individuals within the low-risk group exhibited significantly superior survival outcomes compared to those in the high-risk group (**Figures 6C, D**).

## Discussion

PDTC, an intermediate level of aggression of thyroid malignancies, remains under the investigation of limited research literature. This study was the first to analyze the CS outcomes of these patents, and to develop a novel CS-integrated nomogram model. And the strong predictive performance of this prediction model was verified via a series of evaluation methods. Finally, a risk stratification system was successfully established for a more accurate risk stratification for PDTCs.

In clinical practice, uncertainty about the prognosis of individuals who have survived multiple years postdiagnosis could impede clinical decision-making and potentially impact upon their psychological well-being and overall quality of life (18). In most prior investigations, the prognosis of PDTC was usually analyzed using traditional survival analysis (4, 19–22), but failing to provide more prognosis data for survivors who have survived for several years. The present investigation underscored the pivotal function of the CS in overcoming this limitation of traditional survival analysis. And our novel CS analysis demonstrated a progressive enhancement in the updated real-time survival over time. And it was notable to observe that within the initial stages post-diagnosis, the progressive enhancement in survivor rates overtime was considerable and was concurrent with the steepest trajectory on the survival rate curve. These findings underlined the necessity for increasing the intensity of therapeutic strategies and surveillance during the initial phases following diagnosis for this disease.

Surveillance subsequent to the diagnosis of this disease is an ongoing activity, necessitating the establishment of a dynamic survival estimation tool capable of generating precise predictive information in real-time, enabling tailored surveillance strategies (16). And there are some prognosis prediction models and survival staging systems having been proposed for PDTC patients (5, 20, 23, 24). Jin et al. constructed and validated a nomogram for forecasting the 5- and 10-year cancer-specific survival based on a large cohort with real-world samples (23). Sun et al. also developed a survival staging system providing a more accurate risk stratification for patients with PDTC than the 8th AJCC staging system (5). Nevertheless, despite all these currently published PDTC models, none have offered in real-time, updated prognostication data for survival patients. Consequently, our team developed an innovative CS-nomogram and a risk stratification system to contribute towards filling this existing research gap for PDTCs.

Our nomogram model integrated CS analysis with dynamic survival prediction capability. Moreover, it seamlessly incorporated individualized variables including age at diagnosis, sex, histology type, SEER stage, T stage, N stage M stage, tumor size, coexistence with other malignancy, radiotherapy and marital status, to enhance precise outcome evaluation. On one hand, these variables were identified using the LASSO algorithm; on the other hand, they have demonstrated a correlation with prognosis in clinical practice. Therefore, the predictive model built on these variables is both reasonable and robust. The application of the nomogram model across different clinical scenarios can significantly enhance its practical value. By inputting patients’ clinical characteristics, real-time survival probabilities can be obtained, which can be used to optimize clinical management and decision-making, and facilitate effective communication between doctors and patients. Moreover, this CS-nomogram has potential for providing continually updated prognostic data, empowering clinicians to adapt and refine the patient-tailored surveillance strategy promptly with consideration of evolving risks.

Indeed, the complexity of CS analysis necessitates a relatively large sample size and extended duration for follow-up study, thus, we employed SEER database to carry out this study at a population level. Concerning the performance of the CS-nomogram, it was verified that prediction model established in this study had favorable discriminative ability and accuracy for predicting 10-year CS of PDTC patients. Further DCA analysis demonstrated good clinical utility of the CS-nomogram in clinical practice. These results confirmed that our proposed model could substantially aid clinicians in predicting CS, thereby facilitating them in devising suitable therapeutic interventions for individuals with PDTC.

There are some limitations should be acknowledged in our study. Firstly, this was a retrospective study and potential bias are inevitable. If the SEER database fails to encompass the entire spectrum of patient demographics or treatment contexts, the findings may not apply to populations that differ from those in the database. For example, if specific demographic groups are underrepresented or certain treatment methods are not well-represented, the observed outcomes might not accurately reflect the results for these groups or scenarios. And during sample selection phase, participants with incompletely documented variables were excluded, potentially introducing bias into the results. Secondly, due to the inherent limitation of SEER database, we were unable to procure specific data on patient treatment, including specific radiotherapeutic protocols and surgical type. Thirdly, although our nomogram had been designed and verified within the SEER database, it remains imperative to perform prospective external validations for this predictive model. Finally, it’s imperative to dissect imaging and genetic factors associated with the prognosis of PDTC patients, and further optimize our prognostic nomogram.

## Conclusion

This was the first study to analyze the CS pattern and demonstrated a gradual improvement in CS over time in long-term PDTC survivors. We then successfully developed and validated a novel CS-nomogram for individualized, dynamic, and real-time survival forecasting. And a risk classification system was also constructed for PDTC patient for risk stratification. We further confirmed excellent discriminative ability and potential clinical application of these models.

## Data Availability

The datasets presented in this study can be found in online repositories. The names of the repository/repositories and accession number(s) can be found below: https://seer.cancer.gov/data-software.
